# P017. Ictal cutaneous allodynia does not affect pain perception in patients with migraine: a trigeminal heat stimulation study during interictal period

**DOI:** 10.1186/1129-2377-16-S1-A128

**Published:** 2015-09-28

**Authors:** Antonio Russo, Francesca Conte, Laura Marcuccio, Alfonso Giordano, Gioacchino Tedeschi, Alessandro Tessitore

**Affiliations:** Department of Medical, Surgical, Neurological, Metabolic and Aging Sciences, Second University of Naples, Naples, Italy; MRI Research Center SUN-FISM, Second University of Naples, Naples, Italy; Institute for Diagnosis and Care “Hermitage Capodimonte”, Naples, Italy

## Background

Migraine is a disabling neurological condition characterized by headache attacks, hypersensitivities to visual, auditory, olfactory and somatosensory stimuli, nausea and vomiting. Peripheral and central sensitization of trigeminovascular neurons seem to play a critical role in different aspects of migraine pathophysiology[1]. In the last years, several studies investigated pain thresholds in patients with migraine during both attacks and interictal periods. However, pain perception in patients with migraine has been poorly explored.

## Objective

To investigate perception intensity of trigeminal heat stimulation (THS)[2] in patients with migraine without (MwoA CA-) and with allodynia (MwoA CA+) compared to healthy controls (HC) and correlation with clinical parameters of migraine severity.

## Methods

We enrolled 80 patients with migraine (40 patients with MwoA CA- and 40 patients with MwoA CA+) and 60 HC. THS was performed using the contact heat evoked potential stimulator (CHEPS) at three different intensities: a low-innocuous stimulus at 41°C and two painful heat stimuli at 51° and 53°C (to provide a moderate-noxious and a high-noxious stimulus). Subjects had to verbally rate the intensity perception of the experimental stimulus by means of a numerical rating scale (NRS) ranging from 0 (“no pain”) to 10 (“worst pain imaginable”).

## Results

NRS of pain perception was not significantly different between patients with MwoA (as a group) and HC at any level of experimental stimuli. The absence of significant differences in pain perception was also found between patient groups defined as patients with MwoA CA- and with MwoA CA+ compared to HC, at any level of experimental stimuli.

## Conclusions

In the present study, by using three predefined temperatures, we demonstrated that pain intensity ratings are not significantly different between both CA+ and CA- migraine patients and patients with migraine as a group compared to HC. Previous studies suggested a low heat pain threshold in migraine patients during interictal period[3]. However, it is well known that the pain intensity is different from the pain threshold[4]. Furthermore, we cannot exclude that ictal CA, subtended by central sensitization of trigeminovascular neurons, may revert during interictal period without consequences on pain perception. Central sensitization could become progressively more severe over time and, by an imbalance between the inhibition and the facilitation of pain dynamics, might contribute to chronification phenomena, interictal CA and probably pain perception abnormalities.

Written informed consent to publication was obtained from the patient(s).
